# Addressing the Digital Divide in Health Education: A Systematic Review

**DOI:** 10.7759/cureus.70048

**Published:** 2024-09-23

**Authors:** Anjali Bhoyar, Sunita Vagha, Vedprakash Mishra, Mahima S Agrawal, Seema R Kambala

**Affiliations:** 1 Prosthodontics, Sharad Pawar Dental College and Hospital, Datta Meghe Institute of Higher Education and Research, Wardha, IND; 2 Pathology, Jawaharlal Nehru Medical College, Datta Meghe Institute of Higher Education & Research, Wardha, IND; 3 Physiology, Datta Meghe Institute of Higher Education and Research, Wardha, IND

**Keywords:** digital divide, digital equity, digital health literacy, health education, intervention strategies, telehealth

## Abstract

The disparity in access to essential healthcare resources and services is exacerbated by the digital divide, which presents a significant obstacle to health education. Effective tactics to advance digital equity and provide equitable access to resources for telehealth and digital health are needed to close this gap. Digital databases such as PubMed, MEDLINE, Scopus, Web of Science, and Google Scholar were used to conduct a systematic review. Keywords and Boolean operators including "digital divide," "health education," "digital equity," "telehealth," "digital health literacy," and "strategies" were used in the literature search process. Only peer-reviewed English-language papers that addressed methods for bridging the digital divide in health education were accepted after being screened in accordance with the preset inclusion and exclusion criteria. The results were compiled using a narrative synthesis method after data were retrieved and synthesized with the aid of suitable quality assessment tools. After satisfying the inclusion criteria, seven papers were added to the systematic review. The results emphasized the complexity of the digital divide in health education and provided a range of approaches to mitigate disparities in access to digital health technologies and resources. The importance of digital equality and universal design, continuous intervention evaluation and monitoring, and enduring obstacles to Internet access and healthcare technology availability were among the major themes. This systematic study emphasizes how critical it is to put evidence-based tactics into practice in order to close the digital divide in health education. Through the promotion of universal design principles, ongoing evaluation of treatments, and digital equity, stakeholders can mitigate health disparities, improve population health, and guarantee equitable access to telehealth and digital health services.

## Introduction and background

Digital technology's rapid advancement has revolutionized several industries, including healthcare, by providing formerly unavailable opportunities for medical information access and health education [1]. However, not all populations are able to take advantage of these benefits equally, which leads to the "digital divide" issue. This expression sums up the distinction between those with easy access to digital and information technology and those with little or no access to it [2]. Age, location, education levels, and socioeconomic position are a few of the factors contributing to this divide [3]. The digital divide could significantly impact patient outcomes and the standard of healthcare delivery in the field of health education [4]. Individuals with limited access to digital resources may encounter challenges in promptly obtaining health information, successfully managing their medical issues, or utilizing telehealth services, all of which have grown more crucial in light of the COVID-19 epidemic [5].

Reducing this disparity is essential to guaranteeing fair access to healthcare services and education, which is a basic requirement for enhancing public health outcomes worldwide [6]. Understanding the root causes of the digital divide, identifying the populations most at risk, and putting targeted interventions in place to improve internet access and literacy are necessary to closing the gap [2,6]. Developing inclusive and accessible digital health tools, encouraging digital literacy among marginalized populations, integrating culturally appropriate content that satisfies the needs of diverse communities, and improving infrastructure to provide dependable internet access are just a few of the strategies being used to bridge the digital divide in health education [6]. In order to guarantee that every person has the chance to access essential health information and services in our increasingly digital world, this systematic review aims to evaluate the effectiveness of various strategies that have been used to bridge the digital divide in health education. It also aims to provide a clear understanding of what works, what doesn't, and where additional efforts should be concentrated.

## Review

Method

We adhered to the Preferred Reporting Items for Systematic Reviews and Meta-Analyses (PRISMA) reporting guidelines to ensure transparency, reproducibility, and accuracy in the review process [7]. The PRISMA guidelines provide a structured framework for reporting systematic reviews, facilitating the identification, evaluation, and synthesis of relevant studies. The PECO (Population, Exposure, Comparator, Outcome) protocol for this systematic review is as follows:

P (Population): The review concentrated on people who participated in health education programs or interventions and came from a variety of backgrounds, including those with differing degrees of digital literacy, socioeconomic position, and cultural affiliations.

E (Exposure): The evaluation looked at health education initiatives that attempted to close the digital divide, such as those that promoted health literacy and information access by leveraging digital platforms, online resources, or other creative ways.

C (Comparator): The review examined the effects of digital divide-addressing health education programs and interventions against those that did not, as well as against other strategies for fostering health literacy and information access.

O (Outcome): The assessment assessed how well health education initiatives and programs addressed the digital divide by improving health outcomes, increasing access to health information, and improving health literacy.

Search strategy

We conducted a thorough search of several electronic databases, including Google Scholar, MEDLINE, Scopus, Web of Science, and PubMed. The search utilized keywords and Boolean operators such as "digital divide," "health education," "digital equity," "telehealth," "digital health literacy," and "strategies." The availability of data pertaining to study research concentrated after year 2000, and thus only papers published from years 2000 to 2023 were included in the search. The literature search was performed from January 2023 to February 2024. Reference lists of pertinent papers and systematic reviews were manually searched for further studies.

Inclusion and exclusion criteria

Predetermined inclusion and exclusion criteria were used to screen the articles. All systematic reviews and evidence synthesis scoping reviews, and randomized controlled studies (RCTs) between January 2000 and December 2023, published in English, were included. Studies concentrating on methods to address the digital divide in health professions education were chosen in the inclusion criteria. Only English-language, peer-reviewed articles were taken into consideration for ease of understanding. Published conference abstracts, theses, letters to the editor, non-English publications, editorials, opinion pieces that required additional empirical data, and studies published before January 2000 and after December 2023 were excluded.

A single library was created from the search results obtained from several databases, and duplicate citations were eliminated. After eliminating duplicates, two impartial reviewers went through the titles and abstracts of relevant papers in accordance with the qualifying criteria. Further screening was done to identify research that did not meet the inclusion criteria after irrelevant publications were excluded. In order to locate other relevant publications, we also perused the reference lists of the included articles. Differences among the reviewers were resolved through discourse until a consensus was obtained at every stage of the selection process.

Data extraction protocol

A standardized data extraction form was used to extract data from the included studies. The data that was extracted comprised the author(s), publication year, study design, and sample characteristics, intervention details, outcomes, and key findings related to strategies for addressing the digital divide in health education. The reviewers had discussions to settle any differences in data extraction.

Quality assessment

Depending on the study design, the methodological competence of the studies incorporated was evaluated using the relevant instruments. The Joanna Briggs Institute (JBI) Critical Appraisal Checklist for Analytical Cross-Sectional Studies was utilized for quantitative studies [8]. The JBI Critical Appraisal Checklist for Qualitative Research was employed for qualitative studies. Review articles were assessed using the AMSTAR 2 (AMSTAR, A MeaSurement Tool to Assess systematic Reviews) tool for systematic reviews [9]. Studies were rated based on criteria such as clarity of research aims, sample representativeness, data collection methods, and appropriateness of data analysis.

Data synthesis

A narrative synthesis strategy was used to compile the results from all of the included research. We discovered and aggregated themes and trends pertaining to approaches for closing the digital gap in health education. The main conclusions were drawn, and suggestions for further work and practice were addressed.

Ethical considerations

This systematic review involved the analysis of the existing literature and did not require ethical approval. All data were obtained from publicly available sources, and the confidentiality of study participants was ensured through anonymization in reporting.

Results

**Figure 1:**
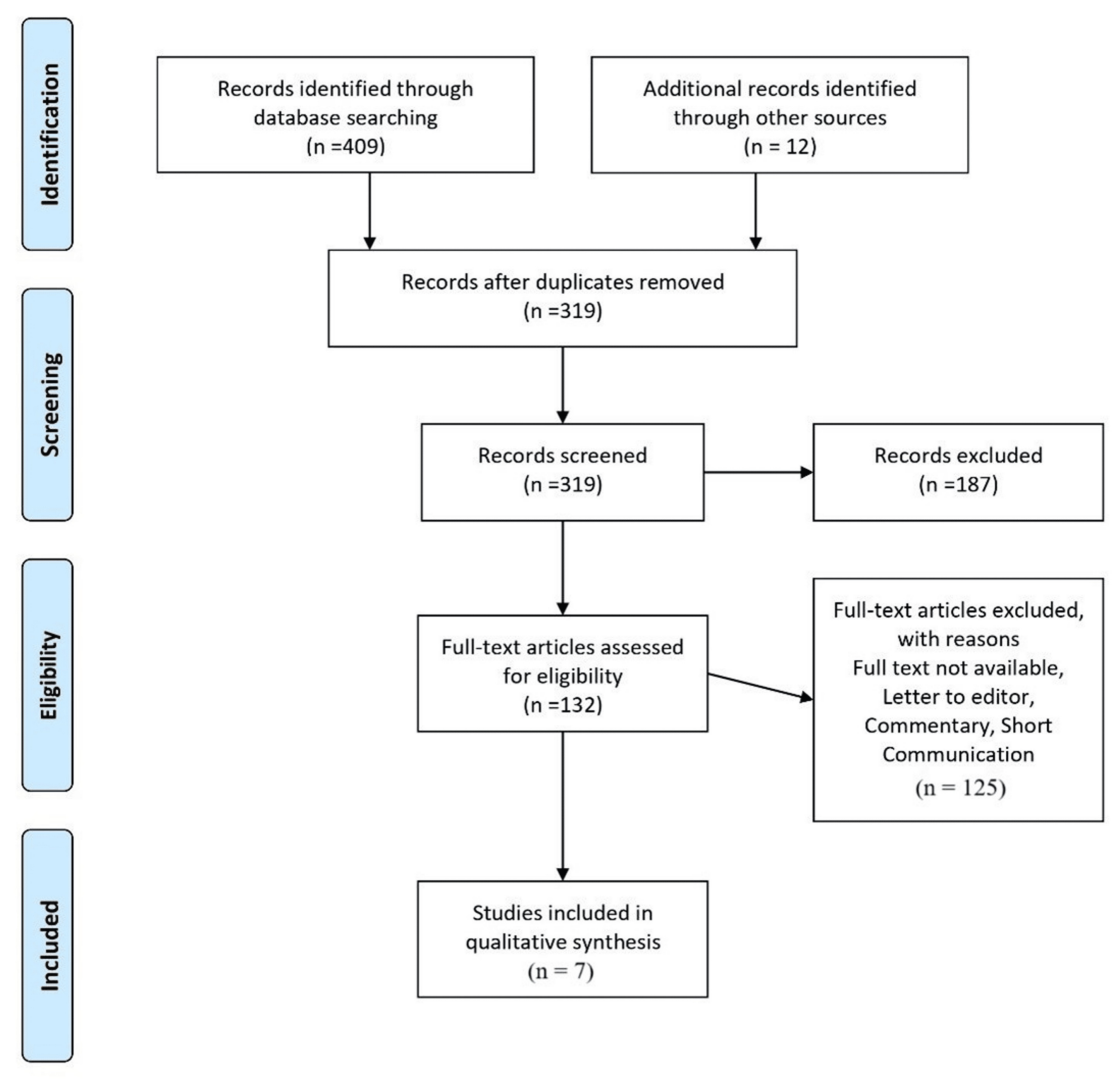
PRISMA flowchart for studies included in the review PRISMA, Preferred Reporting Items for Systematic Reviews and Meta-Analyses

PRISMA guidelines were adhered to in the study selection process for this review, as elucidated through Figure 1. First, a comprehensive search of the literature was conducted, and 409 results were found using database searches. After an additional 12 records were obtained from various sources, there were 421 documents in total. A total of 319 records remained after duplicates were removed, and they were then checked for eligibility. After the screening process, 132 out of the 187 records that were discarded underwent a full-text eligibility review. Following review, 125 full-text items were excluded for a number of reasons, including the fact that part of the text was missing or because they were editorials, brief communications, or letters to the editor. In the end, it was determined that seven studies [10-16] qualified for the qualitative synthesis.

**Figure 2:**
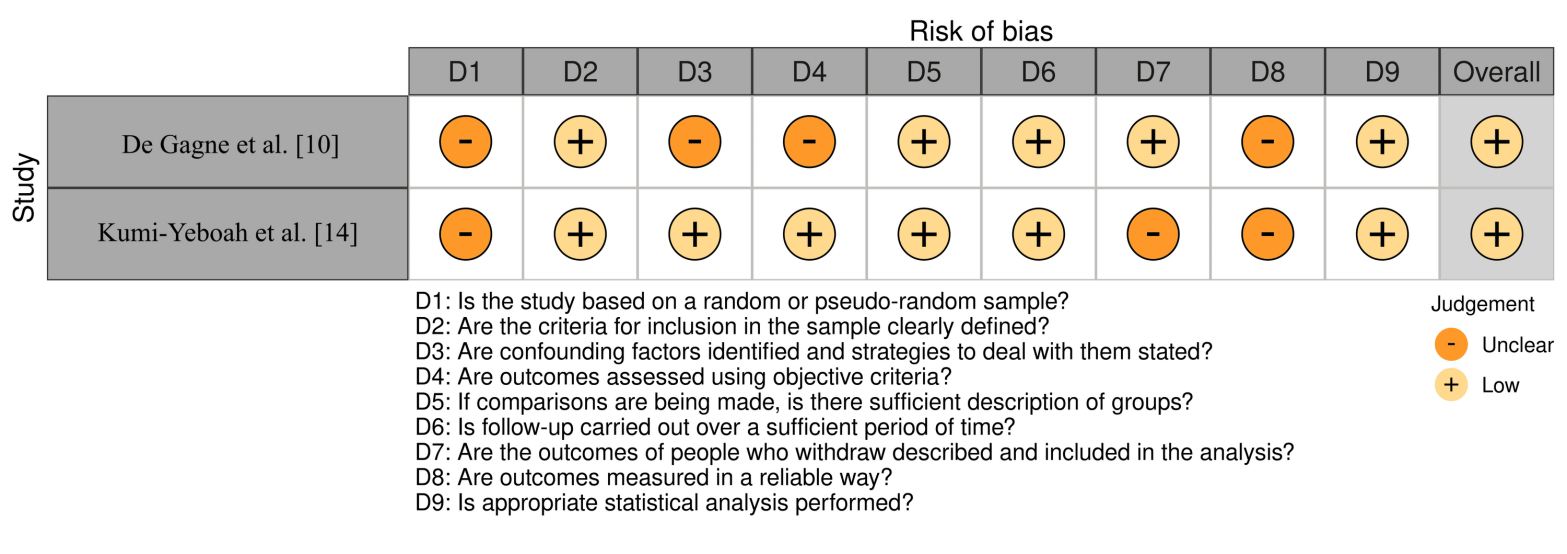
Bias assessment using the JBI appraisal tool JBI, Joanna Briggs Institute

De Gagne et al. and Kumi-Yeboah et al. showed poor to ambiguous scores in several categories, including sampling, confounding factors, outcome assessment, and statistical analysis, using the JBI appraisal checklist (Figure 2) [10,14]. This implies that methodological constraints may make these studies more biased.

**Figure 3:**
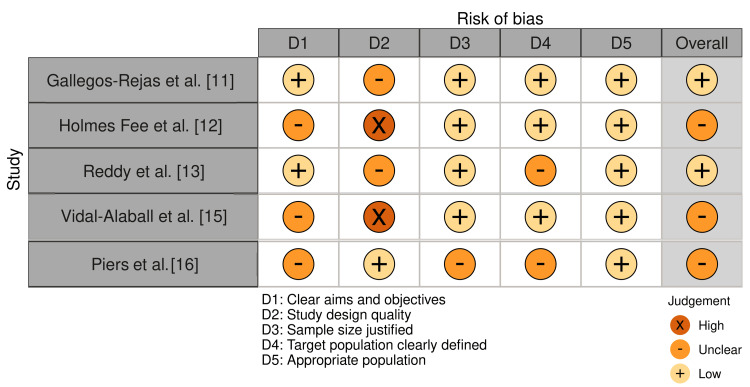
Bias assessment using the AMSTAR 2 tool AMSTAR, A MeaSurement Tool to Assess systematic Reviews

The majority of studies had low scores for research design quality, sample size rationale, target population description, and clear aims and objectives, according to the AMSTAR 2 assessment (Figure 3). For example, low rating was given to the study by Gallegos-Rejas et al. in several domains, suggesting possible biases in their methodological approaches [11]. In contrast, studies by Vidal-Alaball et al. [15] and Holmes Fee et al. [12] scored highly for study design quality but poorly, or were ambiguous, in other categories.

The compilation of the included studies [10-16] is shown in Table 1. Research studies' foci differed noticeably; some focused on underserved populations, such as students pursuing health professions [10], underresourced population groups [11], underinvested localities [12], and Ghanaian higher education institutions [15]. Others took a broader global view, analysing the digital divide in light of the world's population [13]. The evaluations by Vidal-Alaball et al. [15], Holmes Fee et al. [12], and Gallegos-Rejas et al. [11] all emphasized how important it is to address the digital divide in health education, especially for communities that are underfunded and have few resources. Furthermore, there is modest evidence to support the usefulness of digital mental health interventions (DMHIs) in improving mental health outcomes, according to Piers et al.'s review, which focused on children and young people who were socioeconomically and digitally marginalized [16].

**Table 1:** Characteristics of the included studies

Study	Year	Type of Article	Population	Intervention/Topic	Methodology	Digital Divide-Related Findings	Inclusivity/Equity-Related Findings	Conclusion/Implication
De Gagne et al. [10]	2023	Research article	Health profession students	Videoconferencing in education	Systematic review of 9 studies	Technical difficulties, lack of human interactions, and digital divide as barriers to learning	Importance of inclusive pedagogy and digital equity; educators must prioritize digital equity	Videoconferencing has the potential to present challenges related to the digital divide, but also offers learning opportunities
Gallegos-Rejas et al. [11]	2023	Review	Under-resourced population groups	Telehealth access and digital divide	Multi-stakeholder approach	Digital divide as a complex construct; need for practical steps to reduce the digital divide and promote equitable access to telehealth	Practical steps proposed to improve access to telehealth services, including improving digital health literacy and workforce training	Practical steps are needed to reduce the digital divide and promote equitable access to telehealth
Holmes Fee et al. [12]	2023	Review	Underinvested communities	Digital determinants of health (DDOH) solutions	Scoping review of 345 articles	Knowledge gap in the literature on DDOH solutions; 13 underinvested community categories identified	Framework for DDOH assessment proposed; need for nuanced understanding of complex relationships between digital health, equity, and health outcomes	Significant contribution to digital health equity, highlighting the need for a more nuanced understanding of DDOH and its relationships with health outcomes
Reddy et al. [13]	2022	Review	Global population	Role of technology in bridging the digital divide and improving healthcare outcomes	Systematic review	Digital divide hinders equitable access to healthcare services, exacerbated by affordability, lack of internet access, and cultural/health literacy barriers	Addressing social determinants of health and promoting health equity are essential in bridging the digital divide	Technology has a critical role in improving healthcare outcomes and bridging the digital divide; policymakers, healthcare professionals, and stakeholders must collaborate to develop innovative solutions
Kumi-Yeboah et al. [14]	2023	Research article	Higher education institutions (HEIs) in Ghana	Overcoming the digital divide during the COVID-19 pandemic	Qualitative research design, 35 in-depth and semi-structured interviews	Challenges of dealing with the digital divide during the pandemic; lack of access to affordable internet bandwidth	Importance of addressing the digital divide to reduce inequities in teaching and learning; need for inclusive digital infrastructure development	HEIs can address the digital divide by providing training on digital skills and knowledge; understanding the digital divide in sub-Saharan Africa is important
Vidal-Alaball et al. [15]	2023	Review	Healthcare sector	Digital transformation in health care, reducing the digital divide and promoting health equity	Comprehensive review of the literature, including WHO's Global Strategy on Digital Health 2020-2025	Digital divide as a significant barrier to adoption of digital health services; importance of addressing digital determinants of health	Need for cultural and organizational change to promote health equity; importance of equity, accessibility, and affordability in digital health services	Reducing digital divide requires comprehensive approach, including education, infrastructure development, and policy changes; prioritizing vulnerable populations in digital health services is important
Piers et al. [16]	2022	Review	Socioeconomically and digitally marginalized children and young people	Digital mental health interventions (DMHIs) for marginalized youth	Systematic review of 10 studies, convergent integrated approach	Limited evidence supporting DMHIs for improving mental health outcomes; digital divide exacerbates existing health inequalities	Need for tailored DMHIs, consideration of digital divide as a spectrum, and inclusion of communities with unique digital exclusion	There is a need for investments in DMHIs, development of tailored interventions, and addressing the digital divide to promote digital equity

In their investigation of videoconferencing's application in education, De Gagne et al. emphasized technology's capacity to close the digital divide [10]. A multi-stakeholder strategy is required to address the concerns of telehealth access and the digital divide, as highlighted by Gallegos-Rejas et al. in their systematic evaluation of nine strategies [11]. A scoping evaluation of 345 papers was done by Holmes Fee et al. to investigate the implications of digital determinants of health (DDOH) solutions for health promotion and education [12]. Reddy looked into the role of technology in health care and the challenges associated with the global digital divide in healthcare access [13]. Using 35 in-depth and semi-structured interviews, Kumi-Yeboah et al.'s qualitative research study investigated strategies used for closing the digital divide during the COVID-19 pandemic [14]. The World Health Organization's Global Strategy on Digital Health 2020-2025 was included in Vidal-Alaball et al.'s extensive evaluation of the literature, which emphasized the importance of digital transformation in health care, closing the digital gap, and advancing health equity [15]. Using a convergent integrated approach, Piers et al.'s comprehensive review of 10 studies looked at the efficacy of DMHIs for marginalized youth, emphasizing the potential of DMHIs to improve mental health outcomes [16].

According to De Gagne et al., obstacles to learning included the digital divide, a lack of human connections, and technological issues [10]. This highlights the significance of inclusive pedagogy and digital equity. The difficulty of this concept was highlighted by Gallegos-Rejas et al., who underlined the necessity for actionable initiatives to close the digital divide and support fair access to telehealth [11]. Holmes Fee et al. emphasized the need for a nuanced understanding of the linkages between digital health, equality, and health outcomes [12]. They also found a knowledge vacuum in the literature on solutions to address DDOH and provided a framework for DDOH evaluation. According to Reddy et al., hurdles related to health literacy and culture, affordability, and lack of internet connection all increase the digital gap, which makes it more difficult for people to receive healthcare services fairly [13]. In order to lessen disparities in teaching and learning, it is critical to address the digital gap, as demonstrated by Kumi-Yeboah et al.'s study, which highlighted the difficulties in doing so during the epidemic [14]. The thorough assessment by Vidal-Alaball et al. highlighted the significance of equity, accessibility, and affordability in digital health services and stressed the need for organizational and cultural transformation to promote health equity [15]. The systematic review conducted by Piers et al. revealed a paucity of evidence in favor of DMHIs for enhancing mental health outcomes [16]. This underscored the necessity of customised DMHIs, acknowledging the digital divide as a spectrum, and incorporating communities experiencing distinct forms of digital exclusion.

Discussion

The thorough examination of this systematic research highlights the intricacy of the digital divide in health education as well as the need for focused policy implementation to lessen its consequences. Numerous approaches to mitigating the inequalities in access to digital health services and technologies have been identified through synthesis of research findings from several studies. The critical role that digital equity and universal design play in promoting equitable and inclusive access to telehealth services and digital health resources is one of the hottest topics. According to De Gagne et al., in order to guarantee that digital platforms are useable by people with a variety of demands, universal design principles must be followed [10]. The goal of doing this is to reduce the disparities in healthcare service accessibility that exist across various demographic groups.

Gallegos-Rejas et al. suggest collaborating with various stakeholders to mitigate the digital divide in a comparable manner [11]. This policy aims to provide universal access to health care by promoting culturally responsive services and raising awareness of digital health literacy.

The findings underscore the significance of consistently evaluating and monitoring initiatives targeted at bridging the digital gap. Holmes Fee et al. emphasize that reliable research data are necessary to evaluate the efficacy of the tactics used to close the digital divide [12]. This highlights how important it is to implement evidence-based treatments and track their effects on reducing inequities in access to digital health services. But issues still exist in spite of concerted attempts to close the digital gap, especially when it comes to differences in Internet and healthcare technology accessibility.

Reddy et al. emphasized the continued existence of a digital divide in healthcare technology access, emphasizing the need for more efforts to improve technology accessibility for marginalized populations [13]. This calls for a steadfast dedication to putting evidence-based plans into practice, promoting digital equity, and working across sectors to overcome enduring obstacles and guarantee that healthcare services are available to everyone, regardless of location or socioeconomic status (SES).

When we compare the results of our survey to those of previous studies with related goals [17-25], we find several commonalities in the recognition of the digital divide as a major impediment to equitable and accessible health care. Studies have repeatedly shown how differently people utilise and have access to technology, especially when it comes to vulnerable groups like the elderly, people who identify as members of racial or ethnic minorities, and those from lower income backgrounds. It was brought up again how crucial it is to close the digital divide in order to increase health equity and decrease health disparities.

However, the studies' exact focus differed. Adedinsewo et al. [18], for instance, looked into how health equality is impacted by the digital revolution, while Mitchell et al. [17] looked at how elderly individuals use health-related technology. The association between information and communication technologies (ICT) access and the perceived advantages of accessing online health services was investigated by Heponiemi et al. [19], and Fareed et al. [20] investigated cancer sufferers' use of the internet. Notwithstanding these variations, the overall findings demonstrate the necessity of taking focused action to close the digital gap and enhance health equity.

Our study's results are consistent with those of Schroeer et al., who emphasized the drawbacks of digital treatments, such as the possibility of selection bias brought on by the digital divide [21]. Benda et al. emphasized the need of ensuring that everyone has equal access to digital resources and the necessity of taking broadband internet access (BIA) into account as a social predictor of health [23].

Furthermore, investigating how digital technology might enhance health and education was an aim shared by our review and the investigations of Schroeer et al. and Hejna and Seeling [21,22]. While Hejna and Seeling [22] looked at the use of virtual and digital media in connection with the concept of hermeneutic casework in health professional education, Schroeer et al. [21] researched digital formats that allow participation in health promotion and prevention activities in community settings. We also discussed the significance of inclusive and equitable approaches to digital health care in our assessment. On the other hand, differences were discovered in the study designs and emphasis points. While we and Hejna and Seeling [22] carried out systematic reviews, Schroeer et al. [21] used a scoping review design. The specific research questions and objectives of the studies also differed; for example, Hejna and Seeling [22] investigated virtual and digital support in health professional education, Schroeer et al. [21] concentrated on digital formats for community engagement, and our review investigated the digital divide and its impact on health equity. In addition, Benda and associates made a policy-oriented case emphasizing the necessity of government action to close the digital gap and guarantee BIA for everybody [23]. This viewpoint was different from other investigations' more research-focused methodologies.

The existence of a digital gap has been demonstrated by numerous studies on the application and effectiveness of digital health technologies [24]. More precisely, using digital health technologies is more common among those who earn more money and have more education [25]. Remarkably, only 19 randomized controlled studies that specifically examined the efficacy of digital interventions for enhancing physical activity behaviors among various SES levels were included in Western et al.'s systematic review [26]. Furthermore, studies have demonstrated that, in comparison to low-SES groups, high-SES populations typically benefit from digital interventions meant to improve physical activity [26]. There is a dearth of empirical research on this topic, which raises concerns about the possibility that digital health technologies will worsen already-existing health inequities.

Moreover, these research works only look at a small subset of socioeconomic disparity markers. Szinay et al.'s meta-analysis of mobile therapy for weight-related behaviors revealed that most of the included studies concentrated largely on age, gender, education, and ethnicity/race [27]. Less focus has been placed on other significant indicators of inequality that are part of the PROGRESS-Plus framework [28] and include income, occupation, place of residence/living in an urban or rural area, and sexual orientation. It is also important to emphasize that most research on digital health comes from North America, Europe, Australia, and New Zealand and is carried out in Western, educated, industrialized, rich, and democratic (WEIRD) nations [26-28]. This geographic bias highlights the need for a methodical, global, multidisciplinary research of the causes of the digital divide and the influence of social inequality indicators on digital health outcomes. Research of this nature is required to provide fair access to digital health technology and to address health inequities.

Limitations

It is critical to consider the several limitations that our review is subject to. First off, the bulk of the reviewed research focused on the digital gap as a barrier to healthcare accessibility, ignoring the nuances of digital equity and its relationship with social determinants of health. People probably found it challenging to understand the complex relationships between equity, health outcomes, and digital health as a result of this omission. Moreover, the study predominantly utilized a deficit-based technique, emphasizing the deficiency of healthcare services, internet literacy, and accessibility, instead of accentuating the capabilities and adaptability of marginalized communities. This limited perspective may have resulted in missed opportunities to collaborate and develop digital health solutions that consider the unique needs and surroundings of different populations. Moreover, the majority of the reviewed studies were carried out in WEIRD countries, implying that the results might not be a true representation of the global situation regarding digital health. The impression of the digital gap and its effects on health equity may have been influenced by the paucity of research on impoverished populations in low- and middle-income nations as well as WEIRD countries. Additionally, most of the studies employed quantitative methods, which would have obscured the contextual information that qualitative methods may provide that is very essential. It might have been more challenging to comprehend the complex and dynamic linkages between socioeconomic determinants of health, digital health, and health outcomes if more mixed-methods research had been done.

## Conclusions

Our analysis underlines the intricate problems brought up by the digital divide in health education and stresses how critical it is to act quickly to resolve these problems. A review of several studies demonstrated that in order to increase access to telehealth services and other digital health resources, policies that promote digital equality and universal design are required. Moreover, it is imperative to consistently assess and oversee interventions since this ensures the accomplishment of programs aimed at mitigating the digital gap and encourages evidence-based decision-making. However, there are also a number of persistent issues that necessitate continued activism and research to address them, such as disparities in medical equipment accessibility and unequal access to the internet. Stakeholders from all sectors will need to collaborate and use creativity in order to promote health equity and give everyone, regardless of socioeconomic status or geography, equitable access to digital health services.
